# A comprehensive structural, lectin and immunohistochemical characterization of the zebrafish olfactory system

**DOI:** 10.1038/s41598-021-88317-1

**Published:** 2021-04-23

**Authors:** Paula R. Villamayor, Álvaro. J. Arana, Carlos Coppel, Irene Ortiz-Leal, Mateo V. Torres, Pablo Sanchez-Quinteiro, Laura Sánchez

**Affiliations:** 1 Department of Anatomy, Animal Production and Clinical Veterinary Sciences, Faculty of Veterinary, University of Santiago de Compostela, Av Carballo Calero s/n, 27002 Lugo, Spain; 2 Department of Zoology, Genetics and Physical Anthropology, Faculty of Veterinary, University of Santiago de Compostela, Lugo, Spain; 3 Preclinical Animal Models Group, Health Research Institute of Santiago de Compostela (IDIS), Santiago de Compostela, Spain

**Keywords:** Olfactory bulb, Olfactory receptors, Olfactory bulb, Olfactory receptors

## Abstract

Fish chemosensory olfactory receptors allow them to detect a wide range of water-soluble chemicals, that mediate fundamental behaviours. Zebrafish possess a well-developed sense of smell which governs reproduction, appetite, and fear responses. The spatial organization of functional properties within the olfactory epithelium and bulb are comparable to those of mammals, making this species suitable for studies of olfactory differentiation and regeneration and neuronal representation of olfactory information. The advent of genomic techniques has been decisive for the discovery of specific olfactory cell types and the identification of cell populations expressing vomeronasal receptors. These advances have marched ahead of morphological and neurochemical studies. This study aims to fill the existing gap in specific histological, lectin-histochemical and immunohistochemical studies on the olfactory rosette and the olfactory bulb of the zebrafish. Tissue dissection and microdissection techniques were employed, followed by histological staining techniques, lectin-histochemical labelling (UEA, LEA, BSI-B_4_) and immunohistochemistry using antibodies against G proteins subunits αo and αi2, growth-associated protein-43, calbindin, calretinin, glial-fibrillary-acidic-protein and luteinizing-hormone-releasing-hormone. The results obtained enrich the available information on the neurochemical patterns of the zebrafish olfactory system, pointing to a greater complexity than the one currently considered, especially when taking into account the peculiarities of the nonsensory epithelium.

## Introduction

The olfactory subsystems play a fundamental role in the daily life of all animal species^1^, having been extensively studied in mammals, with a fundamental difference between a main olfactory system (MOS) and a vomeronasal or accessory olfactory system (AOS)^2^. While the first one is known for its role in associative behaviours mediated by odorants sensed in the olfactory mucosa^3^, the AOS process innate capabilities mediated by pheromones detected by the vomeronasal organ^4^.

Regarding fish, extensive information has been accumulated in recent decades about their olfactory capabilities^5,6^. Their chemosensory receptors allow them to detect a wide range of water-soluble chemicals, that mediate fundamental behaviours. For instance, nucleotides reveal the freshness of the food^7^, bile acids are implicated in migration to spawning sites^8^, steroids and prostaglandins excreted in urine, trigger reproductive behaviours^9,10^, and injured skin releases alarm pheromones^11^.

In recent decades, zebrafish has become one of the most fruitful model organisms in the field of neurobiology^12,13^. General aspects of its physiology such as external fertilization or rapid development, together with its rapidly accumulating genome sequence data, have made them a suitable model to deepen into genetic engineering and transcriptomic analyses^14–16^. Zebrafish possess a well-developed sense of smell, which governs a variety of behaviours involved in reproduction, appetite, and fear^17^. Moreover, the functional properties within the sensory epithelium and the olfactory bulb (OB) are comparable to those of mammals. Major aspects determined in mammals, as the so-called rule of one receptor-one neuron and the convergence of similar axons in the same glomerulus^18^, are basically preserved in zebrafish^19^. All this makes the zebrafish a model of vertebrate very suitable for studies of olfactory differentiation and regeneration and neuronal representation of olfactory information^20,21^.

The paired nasal cavity of zebrafish is located at the extremity of the snout, between both eyes. Each cavity is composed of an anterior nostril, through which water enters the cavity, and a posterior nostril, through which water exits the nose. The olfactory epithelium (OE) lies between these two nostrils, arranged in several lamellae that converge in a central raphe, forming a cup-shaped structure known as the rosette^22,23^. Lamellae are composed of a continuous sensory area, found in the central and medial region of the rosette, as well as a lateral nonsensory epithelium. The sensory area comprises a characteristic pseudostratified columnar epithelium formed primarily by olfactory sensory neurons (OSNs), as well as basal and supporting cells^24–26^.

Regarding the accessory olfactory system, apart from the isolated case of the *Dipnoi*^27^, all fishes, zebrafish included, lack of a chamber or vomeronasal organ and an accessory OB comparable to those present in amphibians, reptiles or mammals^28^. This led to the conclusion that there is no accessory olfactory system in fish. However, studies of morphological features of the olfactory rosette done in zebrafish^29^ have revealed a complexity that really corresponds to the overlapping and integrated presence of both the main and olfactory systems. Transgenic lines studied by Sato et al.^30^ have unravelled the existence of two segregated neural circuits that originate in the sensory neurons of the OE, each of them featuring specific cell morphology, molecular signatures, and axonal terminations in the OB. Both pathways probably transmit different types of olfactory information (pheromones versus odorants) to higher olfactory centres^31–33^.

In the epithelium of the zebrafish olfactory rosette, five main receptor cell types are differentiated: ciliated, microvillous, crypt, kappe, and pear cells. Ciliated and microvillous are the most numerous neurons and differ from one another for their morphology and relative positions in the OE. The ciliated OSNs are located in the deep layers of the OE, project a long dendrite, and extend several long cilia into the lumen of the rosette cavity. The microvillous OSNs are situated in more superficial layers, bear a short dendrite, and produce short microvilli^34^. The crypt cells are located in the most superficial layer of the OE, have ovoid-shaped cell bodies bearing microvilli and short cilia within the same cell^23,26,35^. The complexity of the zebrafish peripheral OS was proven with the finding of a fourth olfactory sensory neuron population in this species, named kappe neurons for its characteristic shape. These neurons possess microvilli and show a distinct spatial distribution within the OE, similar to, but significantly different from that of crypt neurons^36^. Recently, a small population of OSNs was identified with a pear-shaped morphology and extremely short dendrites, located in the superficial layer of the OE^37^. Finally, scattered among the olfactory sensory neurons are ciliated nonsensory cells, which help to move the mucus covering the OE and basal cells in charge of regenerating the sensory cells^38^.

In addition to these morphological differences, a discrimination among cell types can be clearly made according to their molecular expression profiles^39^. In the zebrafish genome there have been identified 140 OR-type genes^40,41^. The expression of these ORs has previously been observed in the ciliated OSNs^42^, associated with the transduction chain of the G protein-subunit Golf2, which is a direct ortholog of the mammalian Golf protein. Golf has been widely used as a marker of the zebrafish olfactory system^29,43^. 54 V2R-like olfactory C family receptor genes were identified by Alioto and Ngai^44^ and later on the number has been increased up to 60^45^. These receptors are found in the microvillous OSNs^45^. Using genome database mining, Saraiva and Korsching^46^ identified a new family of 6 V1R-type. Whereas half of the fish V1R genes show a multi-exon structure, all mammalian V1R genes possess a single exon structure^47–49^. It has not been clearly demonstrated which type(s) of OSNs express V1R receptors, although the zV1R1 (ORA1) receptor has been characterized in cells belonging to the apical side of the OE^50^ and Oka et al.^51^ found that crypt neurons express a single V1R-type receptor, the ORA4 receptor. Finally, 112 trace-amine associated receptors (TAAR) have been identified in zebrafish^52^.

A comparative analysis between the olfactory transcriptomes of zebrafish and mouse^53^ revealed a high degree of molecular conservation, with orthologs of mouse olfactory cell-specific markers, and all but one of their chemosensory receptor classes expressed in the single zebrafish olfactory organ. All seems to indicate that, despite the remarkable morphological differences between the two classes, Actinopterygii and Mammalia, the molecular mechanisms supporting olfaction in teleost and mammals have similarities despite more than 400 million years of evolutionary divergence.

The large size of zebrafish chemosensory gene families, combined with the high degree of nucleotide identity among their members, make it very difficult to perform comprehensive expression analysis by in-situ hybridization (ISH), Real-time-PCR, or microarray. Although the olfactory system of zebrafish has been subject of sequencing, transcriptomic, and ISH studies, there is a gap regarding immunohistochemical studies that would allow a more comprehensive and structurally precise assessment of its morphofunctional characteristics.

The present study describes the histology, and the lectin-histochemical and immunohistochemical features of the adult zebrafish olfactory rosette and bulb. Three lectins were studied: *Ulex europaeus* agglutinin (UEA), *Bandeiraea simplicifolia* isolectin B_4_ (BSI-B_4_), and *Lycopersicon esculentum* agglutinin (LEA). The immunohistochemical study covered a range of antibodies against the G proteins, Gαi2 and Gαo, the calcium-binding proteins, calbindin (CB) and calretinin (CR), growth-associated protein 43 (GAP-43) glial fibrillary acidic protein (GFAP) and luteinizing hormone-releasing hormone (LHRH).

To our knowledge, 7 of the 10 markers employed in this study have not been previously studied in the zebrafish olfactory system, including the lectins BSI-B_4_, UEA, LEA and the antibodies against Gαi2, CB, GAP-43, and LHRH. These markers have all played key roles in the understanding of the olfactory systems of amphibians, reptiles, and mammals. Therefore, our first aim is to fill the existing gap in the neurochemical characterisation of the olfactory system in zebrafish, which has become a model organism for the study of fields as diverse as developmental biology, cancer, toxicology, and neural regeneration.

Furthermore, our study aimed to address two specific issues. First, in the light of existing studies comparing the olfactory transcriptomes between zebrafish and mouse, which have revealed a high degree of molecular conservation, we aimed to phenotypically characterize two markers of the olfactory sensory transduction chain that are characteristic of the mammalian vomeronasal system, Gαi2 and Gαo, to examine their expression pattern in zebrafish mirrors that in mammals. Second, we aimed to investigate the possible involvement of the nonsensory zone of the olfactory rosette epithelium in chemoreception.

## Material and methods

Ten wild-type 1-year-old zebrafish (*Danio rerio*, wild-type) were used in this study. They were maintained at 28.5 °C in 30 L aquaria at a rate of 1 fish per liter of dechlorinated water, with reverse osmosis purified, and under a light–dark cycle of 14:10. Fishes were euthanized by tricaine overdose (MS-222, Sigma, St. Louis, MO). We followed the ARRIVE guidelines to ensure that all experiments were performed under good conditions. Whole heads were promptly immersed in modified Bouin’s fixative solution. After 24 h, the samples were transferred into 70% ethanol. The samples were not decalcified. In all cases paraffin embedding was used to perform the histological procedures. All samples were cut with a Leica Reichert Jung microtome with a thickness of 4–8 μm. To highlight the different tissue components, we used the following stainings: 1% Alcian Blue (AB) for acid mucopolysaccharides, Nissl staining (1% cresyl violet for 30 min), and Gallego’s Trichrome.

### Gallego’s trichrome

This stain allows for the differentiation of components of the connective tissue. It stains erythrocytes green, muscle fibers and collagen light blue, epithelium and glandular tissue red, bone dark blue and cartilage purple. The protocol used was described in detail in^54^ as follows: sections were first stained with Ziehl acetic fuchsin for 2 min. After several washes they were introduced into formalin-acetic acid solution for 5 min. After two more washes, the sections were finally introduced into picroindigocarmine for 3–5 min.

#### Histochemical and immunohistochemical staining

The histochemical and immunohistochemical protocols followed by the authors have been fully described in previous contributions^54,55^. Briefly:

### Histochemical labelling (HQ) with lectins

We have used (1) a lectin that comes from gorse, the *Ulex europaeus* agglutinin (UEA), α-l-fucose specific, (2) the α-galactose-specific BSI-B_4_ that comes from *Bandeiraea simplicifolia*, and (3) *Lycopersicon esculentum* agglutinin (LEA), coming from tomato with a high affinity for N-acetyl-β-d-glucosamine oligomers (Table 1). These stains selectively recognise the different components of the olfactory and vomeronasal pathways in some species^55^.

**Table 1:** Antibodies and lectins used, with species of elaboration, dilution, manufacturer, and catalogue number.

Ab/lectin*	1st Ab/lectin species and dilution	1st Ab/lectin catalogue number	2nd Ab kit (catalogue number)
Anti-Gαo	Rabbit 1:100	Sta Cruz Biotechnology SC-387	ImmPRESS VR HRP Anti-Rabbit IgG Reagent MP-6401-15
Anti-Gαi2	Rabbit 1:100	Sta Cruz Biotechnology SC-7276	ImmPRESS VR HRP Anti-Rabbit IgG Reagent MP-6401-15
Anti-GFAP	Rabbit 1:400	Dako Z0334	ImmPRESS VR HRP Anti-Rabbit IgG Reagent MP-6401-15
Anti-Calbindin	Rabbit 1:5000	Swant CB38	ImmPRESS VR HRP Anti-Rabbit IgG Reagent MP-6401-15
Anti-GAP-43	Mouse 1:400–1:4000	Sigma G9264	ImmPRESS VR HRP Anti-Mouse IgG Reagent MP-6402-15
Anti-Calretinin	Rabbit 1:5000	Swant 7697	ImmPRESS VR HRP Anti-Rabbit IgG Reagent MP-6401-15
Anti-LHRH	Rabbit 1:500	Fisher Scientific A235481	ImmPRESS VR HRP Anti-Rabbit IgG Reagent MP-6401-15
UEA-I*	1:10	Vector L-1060	Rabbit 1:50 DAKO P289
LEA*	20 μg/ml	Vector B-1175	Vectastain ABC reagent PK-4000
BSI-B_4_*	100 μg/ml	Sigma L-2140	Vectastain ABC reagent PK-4000

Gαo: subunit αo of G protein; Gαi2: subunit αi2 of G protein; OMP: olfactory marker protein; MAP-2: microtubule associated protein-2; GAP-43: growth-associated protein 43; GFAP: glial fibrillary acidic protein; CB: calbindin; CR: calretinin; LHRH: luteinizing hormone-releasing hormone; UEA: *Ulex europaeus* agglutinin; LEA: *Lycopersicum esculentum* agglutinin; BSI-B_4_: *Bandeiraea simplicifolia* isolectin B_4_; HRP: horseradish peroxidase; IgG: Immunoglobulin G; ABC: avidin–biotin-complex.

The lectins employed are indicated by an asterisk (*).

The protocol for the UEA is as follows. (i) blocking the endogenous peroxidase activity of the sample by incubation in 3% H_2_O_2_ solution for 10 min; (ii) incubation for 30 min in 2% bovine serum albumin (BSA), to prevent nonspecific binding; (iii) incubation with the UEA lectin for 1 h; (iv) 3 × 5 min washes in 0.1 M phosphate buffer (PB, pH 7.2), and (v) incubating for 12 h in a peroxidase-conjugated immunoglobulin against the UEA. Finally, (vi) the sections were washed with PB and developed by (vii) incubation in 0.05% diaminobenzidine (DAB) and 0.003% H_2_O_2_ for 5 min.

The protocol for the LEA and BSI-B_4_ begins with the same two steps. Next, (iii) the incubation of the sections was done overnight in biotinylated lectins diluted in 0.5% BSA. The next day, the samples were (iv) 1.5 h incubation in Vectastain ABC reagent (Vector Laboratories, Burlingame, CA, USA). The samples were finally (v) developed in the same DAB solution as the UEA^54^.

### Immunohistochemistry (IHQ) techniques

This protocol also began by (i) blocking the endogenous peroxidase. Then, (ii) non-specific binding was blocked with 2.5% horse normal serum from the ImmPRESS reagent kit Anti-mouse IgG/Anti-rabbit IgG (Vector Laboratories, CA, USA) for 30 min. (iii) The primary antibody was added at the corresponding dilution (Table 1) and incubated overnight. The next day, (iv) the samples were incubated for 20 min with the ImmPRESS VR Polymer HRP Anti-Rabbit IgG Reagent. (v) After rinsing in Tris-buffer (pH 7.61) for 10 min, (vi) the samples were finally developed using DAB in the same way as for the lectins^54,55^.

All immunohistochemical protocols were checked with the appropriate controls. Samples for which the primary antibody was omitted were used as negative controls. Table 2 gives references to previously published use in zebrafish and other fishes of the antibodies here employed against the same antigens.

**Table 2:** Previously published use in fishes olfactory system studies of the antibodies employed in this study against the same proteins.

Antigen	Host	Type, clone	Source	Code	Fish studied	References
Calbindin	Mouse	*Monoclonal*	Swant	300	*Acipenser baeri*	^56^
Calbindin	Mouse	*Monoclonal*	Swant	300	*Polypterus senegalus*	^57^
Calbindin	Mouse	*Monoclonal*	Swant	300	*Polypterus senegalus* *Erpetoichthys calabaricus*	^58^
Calretinin	Rabbit	Polyclonal	Chemicon	AB5054	*Poecilia reticulata*	^59^
Calretinin	Rabbit	Polyclonal	Santa Cruz Biotech	SC-11644	*Danio rerio*	^60^
Calretinin	Rabbit	Polyclonal	Swant	7697	*Danio rerio*	^61^
Calretinin	Rabbit	Polyclonal	Swant	7697	*Danio rerio*	^62^
Calretinin	Rabbit	Polyclonal	Swant	7697	*Salmo trutta fario*	^62^
Calretinin	Rabbit	Polyclonal	Swant	7697	*Psetta máxima*	^63^
Calretinin	Rabbit	Polyclonal	Swant	7697	*Danio rerio*	^29^
Calretinin	Rabbit	Polyclonal	Swant	7697	*Danio rerio*	^64^
GAP-43	Mouse	Monoclonal	Sigma	G9264	*Tilapia mariae*	^65^
GFAP	Mouse	Monoclonal	Sigma	G3893	*Danio rerio*	^66^
GFAP	Mouse	Monoclonal	ZIRC	Zrf -1	*Danio rerio*	^67^
GFAP	Rabbit	Polyclonal	Dako	Z0334	*Oryzias latipes*	^68^
GFAP	Rabbit	Polyclonal	Dako	Z0334	*Danio rerio*	^69^
GFAP	Rabbit	Polyclonal	Dako	Z0334	*Nothobranchius guentheri*	^70^
GFAP	Rabbit	Polyclonal	Dako	Z0334	*Poecilia reticulata* *Carassius auratus*	^71^
GFAP	Rabbit	Polyclonal	Dako	Z0334	*Danio rerio*	^72^
GFAP	Rabbit	Polyclonal	Dako	Z0334	*Astatotilapia burtoni*	^73^
GFAP	Rabbit	Polyclonal	Sigma	G9269	*Danio rerio*	^74^
Gαo	Rabbit	Polyclonal	Santa Cruz Biotech	SC-387	*Tenualosa ilisha*	^75^
Gαo	Rabbit	Polyclonal	Santa Cruz Biotech	SC-387	*Tenualosa ilisha*	^75^
Gαo	Rabbit	Polyclonal	Santa Cruz Biotech	SC-387	*Scyliorhinus canicula*	^76^
Gαo	Rabbit	Polyclonal	Santa Cruz Biotech	SC-387	*Chimaera monstrosa*	^77^
Gαo	Rabbit	Polyclonal	Santa Cruz Biotech	SC-387	*Carassius auratus*	^78^
Gαo	Rabbit	Polyclonal	Santa Cruz Biotech	SC-387	*Scyliorhinus canicula*	^79^
LHRH	Rabbit	Polyclonal	Immuno Nuclear	U-705	*Carassius auratus*	^80^

#### Acquisition of images and digital treatment

Digital images were captured using the Karl Zeiss MRc5 digital camera attached to a Zeiss Axiophot microscope. Adobe Photoshop CS4 (Adobe Systems, San Jose, CA, USA) was used to adjust parameters such as brightness, contrast and balance light levels for presentation in this work. No features of the image were enhanced in any way, moved, or introduced. Some photomicrographs were formed as a mosaic of several photographs merged with an image-stitching software (PTGui Pro, The Netherlands). In no case was this software used to generate images that do not correspond to the actual images presented in the manuscript.

### Ethical approval

The care, use and treatment of zebrafish were performed in agreement with the Animal Care and Use Committee of the University of Santiago de Compostela and the standard protocols of Spain (Directive 2012-63-UE). The protocol was approved by the Animal Care and Use Committee of the University of Santiago de Compostela. Xunta de Galicia Code AE-LU003.

### Informed consent

No human subject was used in this study.

## Results

**Figure 1:**
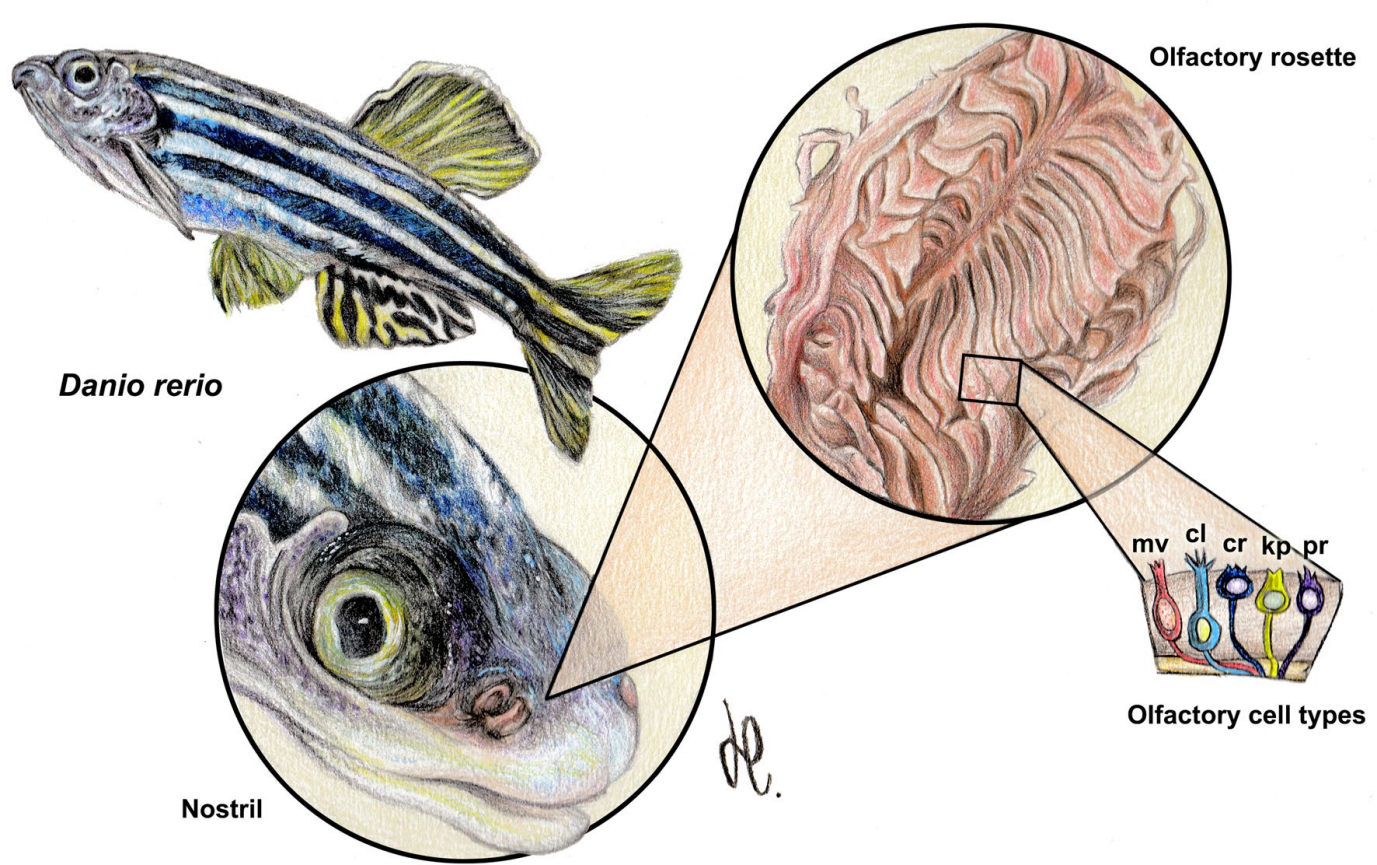
Macroscopic anatomy of the zebrafish olfactory organ. The olfactory rosette occupies in the head an anterodorsal position. The nostrils lie slightly rostral to the eyes and close to the mouth. Five neurosensorial olfactory cell types (OSNs) have been described: microvillous (mv); ciliated (cl); crypt (cr); kappe (kp); and pear (pr) OSNs. Drawing by Helena Reino Piñeiro published under a CC BY open access license.

**Figure 2:**
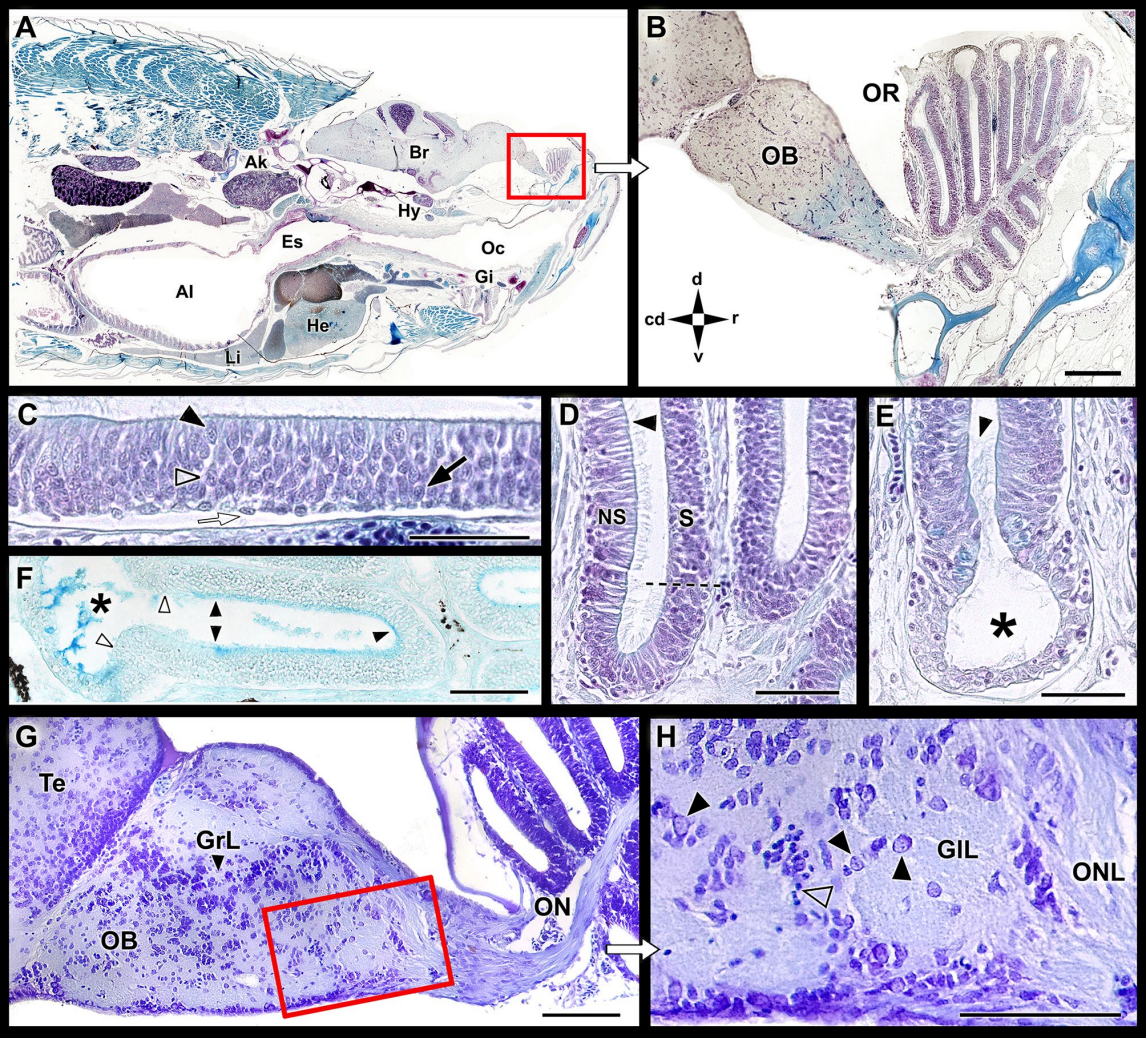
Microscopic anatomy of the zebrafish olfactory system. (**A**) Low power sagittal section of the anterior zebrafish stained with Gallego’s trichrome. (**B**) Higher magnification of the inset in (**A**), showing the olfactory rosette and the olfactory bulb (OB). (**C**) Histological section of the olfactory sensory epithelium stained with Gallego’s trichrome. Black arrowhead, crypt cell; open arrowhead: microvillous cell; back arrow, ciliated cell; white arrow: basal cell. (**D**) Histological section of the medial side of the lamellae. The dotted line demarcates the nonsensory epithelium (NS) of the olfactory epithelium (S). Arrowhead, ciliated cells in the nonsensory epithelium. (**E**) The lateral rim of the lamellae-forming channel-like system (asterisk). Arrowhead, ciliated nonsensory cells. (**F**) Histological section of the lamellae stained by Alcian Blue. The luminal mucociliary complex is restricted to the sensory area (black arrowheads). The nonsensory epithelium border is free from acid mucins (white arrowheads), but Alcian Blue-stained secretions are concentrated inside the channel. (**G**) Sagittal section of the olfactory bulb. (**H**) Inset from (**G**) showing the olfactory nerve layer (ONL) and the glomerular layer (GlL). Black arrowhead, mitral cells; open arrowhead, periglomerular cells. Stains: (**A**–**E**) Gallego’s trichrome; (**F**) Alcian Blue; (**G**,**H**) Nissl stain. Ai, anterior intestine; Ak, anterior kidney; Br, Brain; Es, esophagus; Gi, gilts; GrL, granular layer; He, heart; Hy, hypophysis; Li, liver; Oc, oral cavity; OR, Olfactory rosette; Te, telencephalon; cd, caudal; d, dorsal; r, rostral; v, ventral. Scale bars: 100 µm (**B**,**G**,**H**); 50 µm (**C**–**F**).

The zebrafish olfactory rosette occupies an anterodorsal position, slightly rostral to the orbits (Figs. 1 and 2A, Suppl. Fig. S1A–C). In a parasagittal section at the orbital level, the outlet nostril coincides with a zone of lesser development of the lamellae (Suppl. Fig. S1C). In a sagittal section, which includes the central part of the OB (Fig. 2B, Suppl. Fig. S1B), the rosette can be observed at its maximum expression, divided into two symmetrical zones by the presence of a wide raphe that serves to support the lamellae. The olfactory nerve associated with each lamella constitutes a single branch that reaches the OB from the ventromedial side (Fig. 2G, Suppl. Fig. S1D).

The staining of the sensory pseudostratified epithelium with Gallego’s trichrome reveals the organization and allows for the differentiation of basal cells from the olfactory sensory cells. The three primary olfactory cell types are densely intermingled but can be distinguished by their characteristic shapes and spatial positions: a slender dendrite and a basal soma for ciliated neurons, a rounded cell body and an intermediate soma position for microvillous neurons, and a large globose soma in an apical position for crypt neurons (Fig. 2C). The luminal surface of the nonsensory epithelium is covered by cilia (Fig. 2D,E). Alcian Blue staining shows the presence of acidic mucopolysaccharides on the epithelial surface of the lamella, mainly in the luminal surface of the sensory area, whereas the nonsensory epithelium border is free of acid mucins (Fig. 2F).

The zebrafish OB is diffusely laminated, but its three layers can be identified from the periphery toward the center: olfactory nerve layer, glomerular layer, and granular layer (Fig. 2G,H). The olfactory nerve layer is formed by the axonal endings of olfactory receptor neurons (ORNs) (Fig. 2G). The glomeruli do not resemble the distinct spheres observed in mammals due to the few numbers of periglomerular cells and glial elements found in zebrafish. The cell bodies of the mitral cells are intermingled in the boundaries between the glomerular and granular layers (Fig. 2H). The latest is the deeper layer, which is primarily formed by granule cells (Fig. 2G).

### Immunohistochemical staining of the olfactory rosette

**Figure 3:**
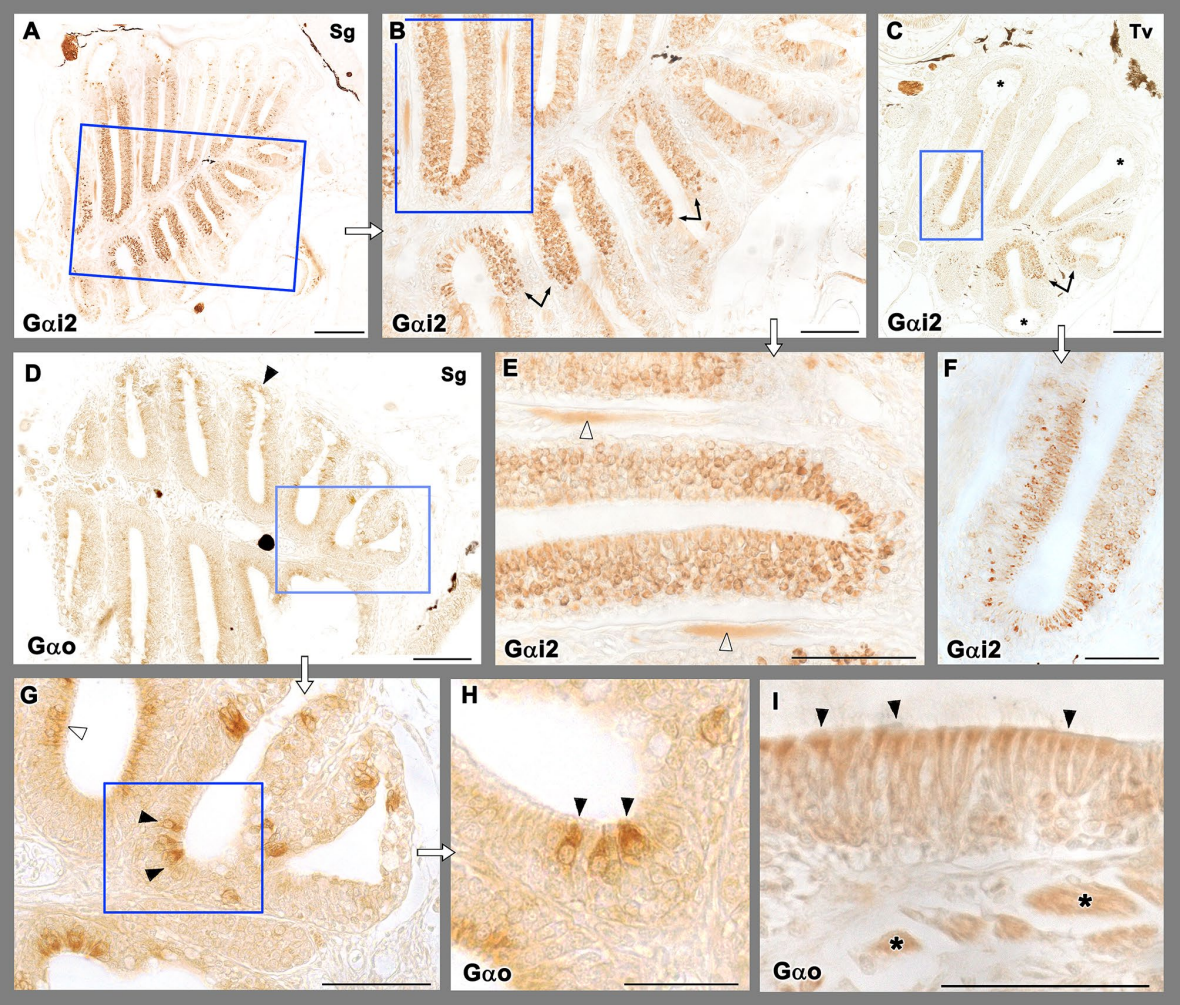
Immunohistochemical study of the olfactory rosette of zebrafish with antibodies against G-proteins. (**A**–**C**,**E**,**F**) Anti-Gαi2 immunolabelling. (**A**) Sagittal sections show a higher number of immunopositive neurons in the internal parts of the rosette, at both sides of the median raphe. (**B**) Higher magnification of the inset in (**A**). The transition between both the nonsensory and sensory epithelium is straight (arrows). (**C**) Transverse sections show how the lateral rim of the lamella (asterisks) lacks of anti-Gαi2 immunolabelling. (**E**) Inset in (**B**) showing the immunopositive olfactory nerves (white arrowheads). (**F**) Inset in C shows how the basal part of the neuroepithelium lacks immunopositive cells. (**D**,**G**–**I**) Anti-Gαo immunolabelling. (**D**) The immunoreactivity was present diffusely in apical neurons, as it is shown by open arrowheads in the inset (**G**). Additionally, more isolated big and oval receptor neurons showed immunoreactivity (black arrowheads) (**H**, inset in **G**). (**I**) The processes in the nonsensory epithelium (arrowheads) and the branches of the olfactory nerves (asterisks) are also immunopositive. Scale bar: 100 μm (**A**,**C**,**D**); 50 μm (**B**,**E**–**I**).

The immunohistochemical study with anti-Gαi2 and anti-Gαo produce two differentiated patterns (Fig. 3). Intense immunoreactivity was noticed in the central and medial portions of the OE when employing Anti-Gαi2 (Fig. 3A–C,E,F). The immunopositive cells are distributed across the entire thickness of the epithelium, but appear mostly concentrated in its superficial half (Fig. 3F). Contrastly, anti-Gαo produces a diffuse immunoreactivity circumscribed to cells present on the apical surface of the OE (Fig. 3D,G–I). Interestingly, we also found large isolated cells with stronger anti-Gαo immunopositivity in the OE (Fig. 3H). Additionally, the anti-Gαo labelling also stains the apical surface of the nonsensory area of the hair cells (Fig. 3G). Both markers, anti-Gαi2 and anti-Gαo label the olfactory nerves (Fig. 3E,I).

**Figure 4:**
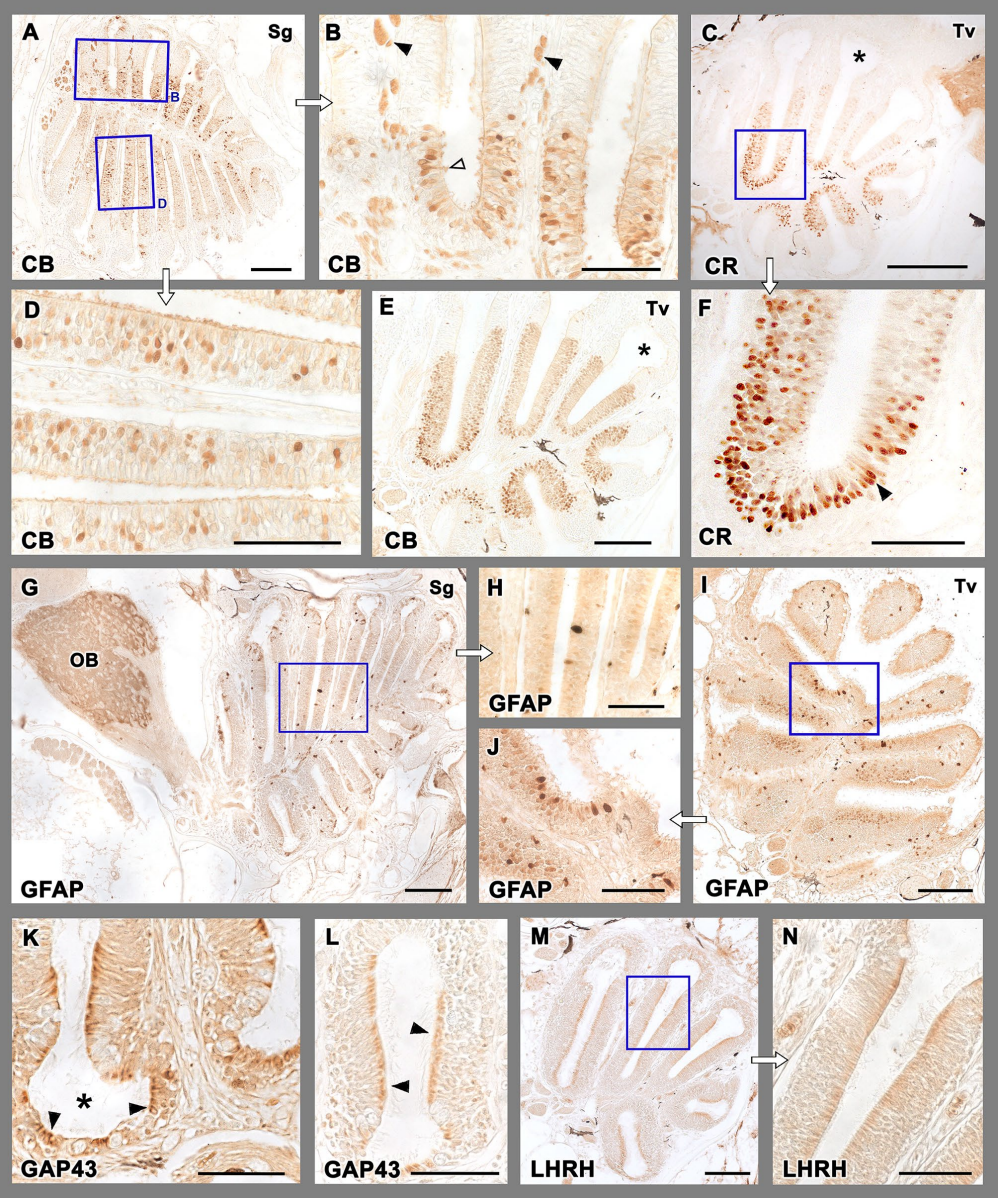
Immunohistochemical study of the olfactory rosette of zebrafish. (**A**,**B**,**D**,**E**) Anti-Calbindin labelling. (**A**) A sagittal section of the olfactory rosette shows labelling in a neuronal subpopulation distributed widely in the rosettes. Insets in (**B**) and (**D**) show the immunolabelling mostly located in the deeper part of the epithelium. In (**B**), neuroephitelial cells in the superficial layer (open arrowhead) and branches of olfactory nerves (black arrowheads) are intensely labelled. A transverse section (**E**) shows a similar pattern. The nonsensory epithelium (asterisk) is immunonegative. (**C**,**F**) Anti-Calretinin immunolabelling produces a stronger labelling of neuroepithelial cells, mostly concentrated in the medial part of the lamellae, and their deeper layers, whereas the nonsensory cripts (asterisk) are not immunolabelled. The neuroepithelial cells are mainly distributed in deeper layers (black arrowheads in **F**), and very rarely the superficial cells are lightly immunolabelled. (**G**–**J**) Anti-GFAP immunolabels isolated big cell bodies in the apical part of the epithelium. They appear in both sagittal (**G**) and transverse (**I**) sections. Insets are shown in (**H**) and (**J**), respectively. Additionally, in the olfactory bulb the antibody anti-GFAP labels the whole glomerular layer (OB). (**K**,**L**) Anti-GAP-43 immunolabelling is located in the apical part of the nonsensory epithelium (black arrowheads in **K**) and in individual cell bodies (black arrowheads in **L**) in the crypts (asterisk). (**M**,**N**) Anti-LHRH produces a light immunolabelling, mainly located in the cell processes of the nonsensory epithelium. Scale bars: 100 μm (**A**,**C**–**E**,**G**,**I**–**M**); 50 μm (**B**,**F**,**H**,**N**).

The results of the immunohistochemical study with anti-calbindin (anti-CB) and anti-calretinin (anti-CR) are depicted in Fig. 4A,B,D,E and C,F respectively. Both markers label a subpopulation of olfactory sensory neurons. Sagittal and transverse sections of the rosette show how the anti-CB inmmunolabelling is mostly located in the deeper part of the neurosensorial epithelium (Fig. 4D), whereas the nonsensory epithelium is Anti-CB immunonegative (Fig. 4B). Anti-CR immunolabelling produces a stronger labelling than anti-CB in neuroepithelial cells, which is mostly concentrated in the medial part of the lamellae (Fig. 4C), and their deeper neuroepithelial layers (Fig. 4F). Very rarely superficial cells are immunolabelled. The nonsensory cripts do not show anti-CR labelling.

Anti-GFAP immunolabels isolated big cell bodies in the apical part of the whole OE (Fig. 4G–J). These cell bodies are present in both sagittal and transverse sections. Anti-GAP-43 immunolabelling is located in the apical part of the nonsensory epithelium and in individual cell bodies in the crypts. (Fig. 4K,L). Anti-LHRH produces a light immunolabelling, mainly located in the cell processes of both the sensory and nonsensory epithelia (Fig. 4M,N).

### Lectin histochemical staining of the olfactory rosette

**Figure 5:**
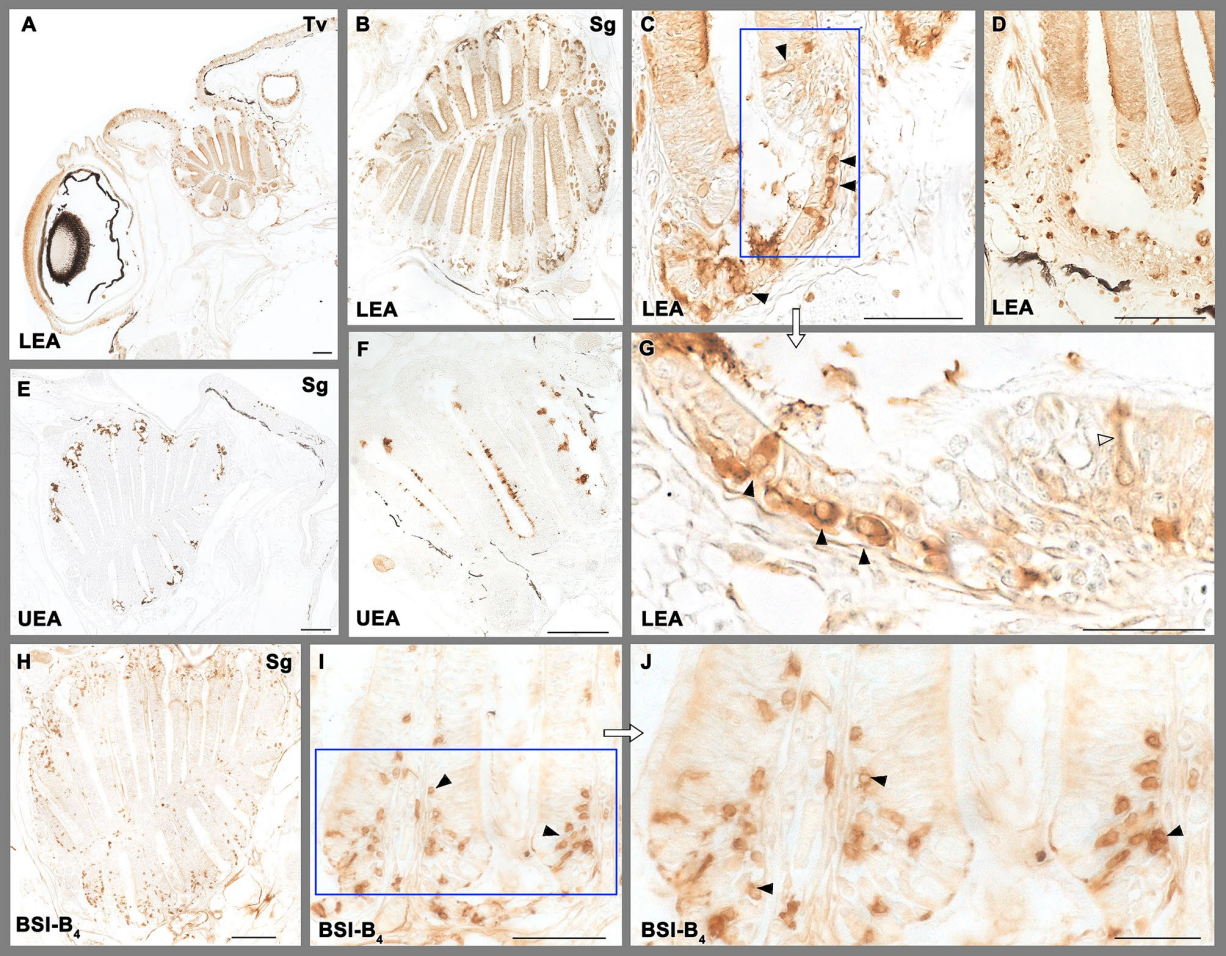
Lectin histochemical staining of the olfactory rosette. (**A**–**D**,**G**) LEA marks intensely the olfactory epithelium dividing it clearly from the unstained nonsensory epithelium in both transverse (**A**) and sagittal (**B**) sections. In the nonsensory epithelium (**C**,**D**,**G**) it labels big oval neuron-like somas (black arrowheads) in the crypts. Thin dendritic processes can be clearly appreciated (white arrowhead in **G**). (**E**,**F**) UEA labels secretory material, mainly in the crypts, but also in the sensory epithelium. (**H**–**J**) BSI-B_4_ labels neuron-like cells in the nonsensory epithelium (arrowheads), including its crypts. Scale bars: 100 µm (**A**,**B**,**D**,**E**,**H**); 50 µm (**C**,**F**,**I**); 25 µm (**G**,**J**).

The three lectins employed in this study labelled the olfactory system following individual patterns (Fig. 5). The labelling with LEA of the whole OE clearly delineates the limits between the olfactory and the nonsensory epithelium -this latter unstained (Fig. 5A,B). Additionally, big oval neuron-like cell bodies in the crypts of the nonsensory epithelium are also stained, and in some of them thin dendritic processes can be clearly appreciated (Fig. 5C,D,G). UEA only labelled secretory material, mainly in the crypts, but also in the sensory epithelium but in lesser extent (Fig. 5E,F). BSI-B_4_ marks individual cells in the crypts and in the nonsensory part of the lamella and very occasionally cells belonging to the sensory part of the OE. In all cases they are scattered cells, smaller than those marked by LEA (Fig. 5H–J).

### Immunohistochemical and lectin histochemical staining of the olfactory bulb

**Figure 6:**
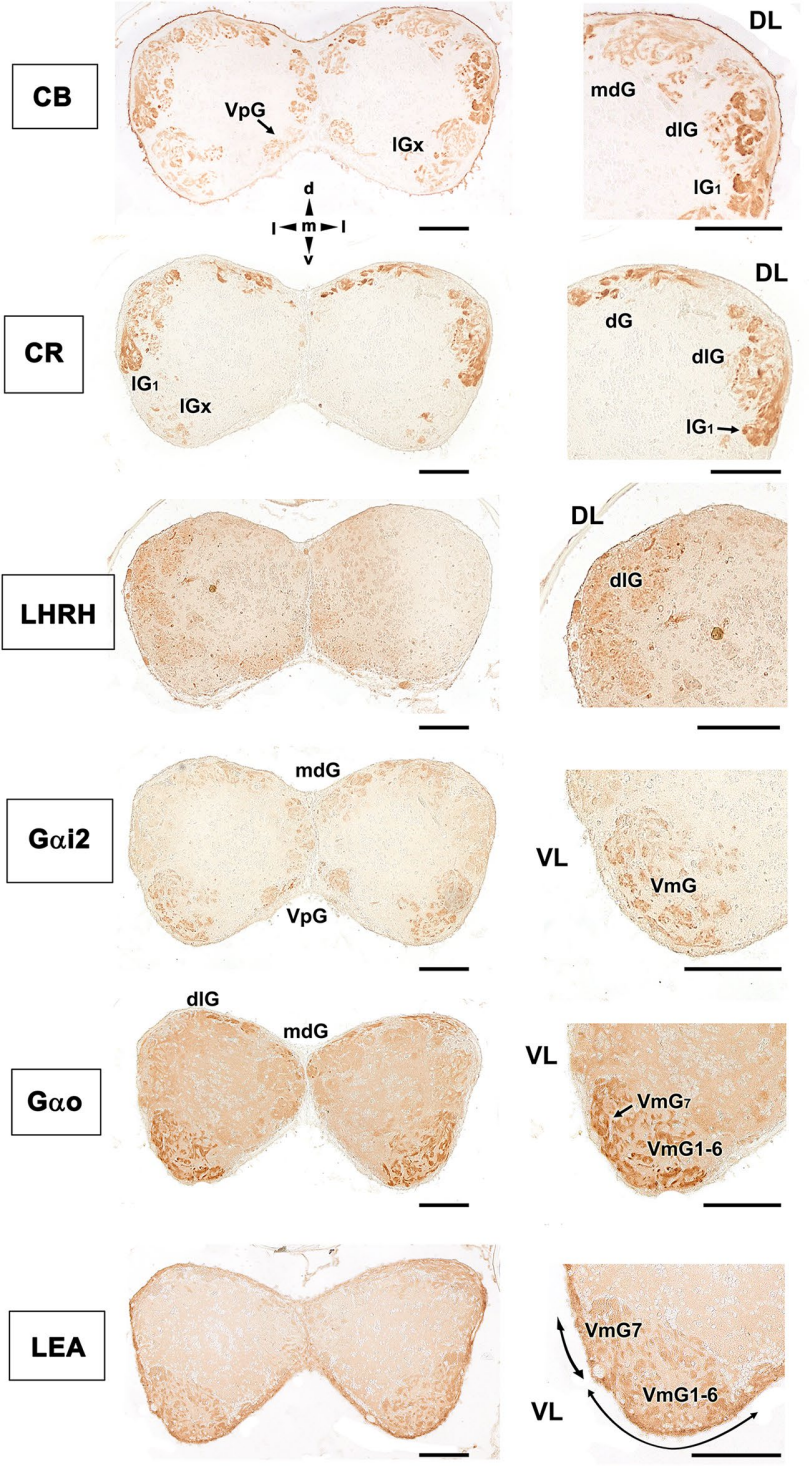
Immunohistochemical and histochemical labelling of the zebrafish olfactory bulb. Transverse sections through the central area of the olfactory bulb showing immunoreactivity with the antibodies anti-CB, anti-CR, anti-LHRH, anti-Gαi2, anti-Gαo and LEA lectin labelling. Calcium-binding proteins (CB and CR) are both mainly expressed in the dorsal (dG), dorsolateral (dlG) and lateral (lGx, lG_1_) glomeruli. Additionally, calbindin is also expressed in glomeruli belonging to the mediodorsal part of the bulb (mdG) and, with less intensity, in the ventral posterior area (VpG). LHRH is expressed in the dorsolateral glomeruli. Both anti G-proteins show a similar pattern, with an intense labelling in the ventromedial glomerular (VmG) area, stronger in the case of Gαo. Additionally, anti-Gαi2 marks glomeruli belonging to the ventral posterior cluster (VpG). LEA labelling is mainly circumscribed to the ventral region. DL, dorsolateral; VL, ventrolateral; dG, dorsal glomeruli; dlG, dorsolateral glomeruli; lG_1_, lateral glomerulus 1; lGx, lateral cluster of glomeruli; mdG, mediodorsal glomeruli; VmG_1-6_, ventromedial glomeruli 1–6; VmG_7,_ ventromedial glomerulus 7; VpG, ventral posterior glomeruli; d, dorsal; l, lateral; m, medial; v, ventral. Scale bars: 100 µm.

The immunohistochemical labelling of the olfactory bulb (Fig. 6) primarily reveals two patterns. The labelling for the calcium-binding proteins (CB and CR) is concentrated in the dorsal and lateral regions. Using the terminology established by Braubach et al.^61,81^, calcium-binding proteins were identified in the dorsal glomeruli (dG), clusters of the dorsolateral (dlG) and lateral glomeruli (lGx), and the large lateral glomerulus 1 (lG_1_). Additionally, anti-calbindin stained glomeruli belonging to the mediodorsal (mdG) and the ventromedial parts of the bulb were stained with reduced intensity (ventral posterior glomeruli, VpG). Both G protein antibodies showed similar patterns, with a higher concentration of labelling in the ventromedial glomerular cluster 1–6 (VmG_1-6_) and the large ventromedial glomerulus 7 (VmG_7_), which appeared stronger for anti-Gαo. Both G proteins faintly stained the mediodorsal glomeruli (mdG), and Gαi2 marks glomeruli belonging to the ventral posterior cluster (VpG). Finally, LEA exemplifies a second pattern, in which the labelling was mainly circumscribed to the ventral area. This lectin also labels the dorsolateral area but with less intensity. LHRH is expressed primarily in the dorsolateral glomeruli (dlG). Anti-GAP-43 and the other two lectins examined in this study did not show significant results in the OB.

## Discussion

Under an appearance of simplicity, in recent decades the olfactory system of fish has revealed a very high structural complexity, according to cell types and receptors involved. The advent of genomic techniques such as RT-PCR, ISH, genomic and transcriptomic analyses, has been decisive for the discovery in fish of specific olfactory sensorial cell types such as kappe or crypt cells. These techniques have also played a significant role in the identification and characterization of large cell populations expressing vomeronasal receptors^42,44^, thus putting an end to the longstanding controversy about the existence of an AOS in fish^32^.

In zebrafish, genomic advances have been so rapid that they have unavoidably marched ahead of morphological and neurochemical studies. Instead, studies in mammals, have formed a solid basis on which to build knowledge of the olfactory and vomeronasal systems^82,83^. For this reason, there is a lack of specific histological, lectin-histochemical and immunohistochemical studies of the olfactory rosette and OB of the zebrafish. To discuss our results, we must therefore contextualize them in a higher taxonomic order including other fish families.

Although the presence of a vomeronasal organ is a tetrapod evolutive innovation, the vomeronasal receptor genes have been identified in fish and even in the lamprey^84^. Taking into account that each vomeronasal type receptor, V1R and V2R, is associated in mammals to a unique G protein, Gαi2 and Gαo respectively, the present study aimed to determine whether there is a correlation between such G-proteins and the zebrafish olfactory cell morphology. It is known from the literature that the receptor molecules and the G-protein specific for each receptor are detectable not only in the dendritic process of the neuroreceptor cell, but also along the axons and their termination in the glomeruli of the OB^85^. For this reason, we have extended the study of these molecules of the chain transduction to the olfactory bulb.

Regarding Gαo, Hansen et al.^78^ correlated in goldfish *Carassius auratus* the receptor cell morphology and the cell types distribution, with the expression of G-proteins, demonstrating that anti-Gαo immunoreactivity was present on microvillar ORNs located in the upper half of the OE. This happens similarly in the Gαo neurons identified by us in the zebrafish, pointing to the reliability of anti-Gαo as a marker of microvillar V2R-like cells in this species. Other studies in different fish species, such is the case of Chondrichthyes, support this view. Thus, immunohistochemical studies of G protein α subunits in the olfactory organ of *Scyliorhinus canicular* (Elasmobranch) and *Chimaera monstrosa* (Holocephali) found the presence of Gαo in virtually all ORNs, which was consistent with the presence of V2Rs^76,77^.

In our case, additionally to the profuse microvillar Gαo-positive neurons, we have also found a subpopulation of large, oval-shaped Gαo positive cells, always located on the apical surface of the OE. Although their morphology is reminiscent of crypt cells, the study by Ahuja et al.^36^ in zebrafish demonstrated that these cells constitute a new olfactory cell type, the kappe cells. Their immunofluorescence study showed that kappe neurons are identified by their anti-Gαo immunoreactivity, demonstrating a scattered spatial distribution within the OE, similar to, but significantly different from that of crypt neurons.

Ahuja et al.^36^ also found that kappe neurons project to a single identified target glomerulus within the OB, belonging to the mediodorsal cluster. This observation coincides with the anti-Gαo pattern of labelling found by us in the OB, showing immunopositivity to Gαo in the mediodorsal part of the bulb. We also found an intense immunoreactivity to anti-Gαo in the cluster of ventromedial glomeruli 1–6 (VmG_1-6_) and the large ventromedial glomerulus 7 (VmG_7_), an area which has been attributed to an important projection of fibers from microvillar cells^30,85^. However, this observation contrasts with that obtained by Braubach et al., who did not find any anti-Gαo positivity in the ventral glomeruli and only detected Gαo in the mediodorsal glomerulus 5 (mdG_5_)^61^.

Our observations confirm the validity of the anti-Gαo antibody as a reliable marker of V2R-like receptor cells in zebrafish, pointing to the presence in zebrafish of a large population of microvillar and kappe cells whose transduction chain is analogous to that present in mammals V2R vomeronasal cells. Therefore, this appears to be an ancient trait conserved through the vertebrate evolution^78^.

Regarding crypt olfactory receptor cells, none of the antibodies and lectins employed by us have labelled specifically these cells. Nonetheless, the study by Catania et al.^86^ characterized immunohistochemically these crypt cells in zebrafish employing an antibody against the neurotrophin receptor Trk-A.

To our knowledge, the inhibitory subunit Gαi2 has not been studied in the fish olfactory system. This fact is surprising since Gαi2 is part of the vomeronasal V1R receptor transduction chain in mammals. Mammalian Vmn1r genes show a rather dynamic evolution, in striking contrast to the highly conserved fish orthologous, the ORA gene family. In zebrafish, six ORA receptors have been identified^46^, and only in one of them, the ORA4, the precise location of its expression has been studied. Thus, ORA4 receptor was found to be expressed in crypt neurons, but not associated with Gαi2 but with the inhibitory G protein, Gi1b^51^. As for its ligands, it is only known that the ORA1 gene recognizes with high specificity and sensitivity the 4-hydroxyphenylacetic acid^87^, which might function as a pheromone for reproductive behaviour in zebrafish. ORA1 is ancestral to mammalian V1Rs, and its putative function as pheromone receptor is reminiscent of the role of several mammalian V1Rs as pheromone receptors.

The anti-Gαi2 pattern of labelling is very different from that found with anti-Gαo, as it covers a wider thickness of the epithelium, although it rarely reaches the deepest cell layers. Moreover, unlike anti-Gαo immunolabelling, it comprises the entire extension of the olfactory epithelium clearly demarcating it from the nonsensory epithelium. The immunohistochemistry of the olfactory bulb using anti-Gαi2 results in the labelling of a huge glomeruli subpopulation, mainly belonging to the ventromedial and ventral posterior clusters, confirming that the Gαi2-positive cells in the olfactory rosette are sensory neurons that convey information to the brain. If ORA receptors coincide with the V1Rs in having the protein subunit Gαi2 in their transduction chain, it is surprising that such a small number of receptors are expressed on such a high number of olfactory cells as those detected in our zebrafish olfactory rosette and bulb immunolabelling.

Calcium-binding proteins contribute to calcium homeostasis by buffering the intracellular free calcium concentration^88^. Both CR and CB protect sensory neurons against calcium increases during periods of high frequency discharge as well as in pathological conditions^89^. Moreover, calretinin and calbindin immunoreactive (CR-ir and CB-ir, respectively) neurons in the cerebral cortex are resistant to degenerative processes in Alzheimer’s disease^90^.

The distribution of CR in the olfactory system of the zebrafish was investigated for the first time by Castro et al.^62^, by using immunocytochemical techniques. Our CR immunoreactivity coincide essentially with their own observations. Accordingly, it is remarkable the presence of numerous CR-ir bipolar cells in the neuroepithelium and an intense immunopositivity in the olfactory nerve. Parisi et al.^64^ performed immunofluorescence against CR on zebrafish crypt cells, finding immunolabelling in the OE, primarily in the intermediate cells, but also in the superficial layer. Morphologically, immunopositive cells resembled to them both microvillous and crypt cells. However, our light microscopy anti-CR immunopositive cells featured a slender dendrite and an elongated soma; a morphology closer to olfactory than to microvillous cells. Our observations agree with the immunoelectron microscopy investigation by Gayoso et al.^29^ who found a consistent anti-CR immunopositivity in the ciliated cells and only very rarely in the microvillar cells. Additionally, neither Castro et al.^62^, Gayoso et al.^29^ nor us have found anti-CR immunopositive crypt cells.

Calretinin immunolabelling of the olfactory rosette has been used in toxicity studies examining the effects of various chemicals, such as copper, zinc, urea, and detergents^91–94^. Although the labelling patterns observed in the control individuals of these studies are similar to ours, in some cases^93,94^, higher staining intensity was obtained in these studies than was obtained in our samples. Both of these toxicity studies were performed using samples decalcified with EDTA, an agent that can act as an antigenic retrieval agent. In addition, these studies have used a polyclonal antibody from a different source than ours. Therefore, the results of these studies should not be compared to our results in terms of intensity. The reduced staining intensity observed in our study allows for the better discernment of cell morphology; however, to facilitate comparisons between morphometric studies, the standardization of staining methods should be considered. Finally, studies in other fish species such as the one carried out in the olfactory rosette of guppy^59^ have proved similar observations to those found by us in zebrafish.

In the OB, Castro et al.^62^ and Braubach et al.^61^ each presented comprehensive studies of the entire olfactory glomeruli population and found that the dorsal, dorsolateral, and lateral glomeruli clusters, the large lateral glomerulus 1, and the ventromedial glomerular fields exhibited strong anti-CR labelling, whereas the dorsomedial area exhibited only faintly CR-ir fibers. Our observations are mostly comparable to those found by them, with the exception that we did not find immunopositive ventromedial glomeruli, likely due to a difference in the levels chosen for our study.

Regarding the expression of calbindin in the olfactory system of the zebrafish, there is a lack of information. However, there have been studies in other fish species such as the chondrostean, *Acipenser baerii*^56^ and the cladistian fish *Polypterus senegalus*^57^, in both cases finding a very faint expression in the olfactory rosette. Our study shows a very high expression of calbindin in the zebrafish olfactory organ, which is accompanied by a parallel expression in the OB. The immunolabelling is comparable to that produced by anti-calretinin, but wider in the case of calbindin, as it is extensible to the ventral posterior glomeruli and the mediodorsal glomeruli, bigger than the dorsal and dorsolateral glomeruli.

The expression of GFAP has been very little studied in the olfactory system of fish. Notwithstanding, there is a specific study in zebrafish by Lazzari et al.^72^ about olfactory ensheathing cells (OECs) with employed different markers (antibodies against GFAP, S100, NCAM, p75). OECs are unique glial cells with axonal growth-promoting properties, involved in the regenerating capability of ORNs throughout life. These cells sustain the continuous axon extension and successful topographic targeting of the olfactory receptor neurons. They are present in the OE and the OB and are also expressed along the entire length of the olfactory nerve. Lazzari et al.^72^ reported slight immunostaining in the OE, moderate staining in the OB olfactory nerve layer, and faint positivity in both the glomerular layer and the inner bulbar zone of zebrafish. Our study identified stronger labelling in both the olfactory nerve and the glomerular layers of the bulb. Although we have used the same fixative and commercial antibody as those used by Lazzari et al.^72^, their samples were treated for decalcification by EDTA, whereas we did not subject the tissue to any types of chelating treatment. This difference in sample treatment might explain the observed differences in the immunolabelling intensity observed between the two studies. Other studies performed in zebrafish have reported an anti-GFAP immunolabelling pattern in the OB similar to ours. For example, Byrd and Brunjes^25^ reported that the olfactory nerve and glomerular layers feature predominant GFAP immunoreactivity, and Scheib and Byrd-Jacobs^95^ reported numerous processes concentrated in the nerve and glomerular layers (GL). Additionally to the previous reports, we found the presence of immunopositive cells in the apical surface of the epithelium, which vary in shape and size, but they are predominantly globose. The meaning of such immunopositive cell bodies, previously undescribed, should be further studied.

Anti-LHRH has been used in fish and mammals to characterize the terminal nerve^80^; a ganglionated extrabulbar nerve, independent of the olfactory nerve, but close enough to be identified by classical histological methods. Its function is uncertain, although its fibres facilitate migration of LHRH cells to the hypothalamus, thus participating in the development of the hypothalamic-gonadal axis^96^. Although extrabulbar elements have been characterized in the forebrain of the zebrafish^29^, they differed from the terminal nerve as they were not immunoreactive to the most widely employed marker for this nerve, FMRFamide^97^. We were not able to identify LHRH fibers in the olfactory rosette, but we observed immunoreactivity in neuronal elements of the dorsolateral OB. This result is consistent with that observed in fish by Münz and Class^98^ and mammals by Witkin and Silverman^99^.

Histochemical labelling with lectins has been widely used in fish, but to a lesser extent in the olfactory system, and with only a few references to the specific case of zebrafish. UEA-I, specific for l-fucose, has been studied in trout^100^ finding intense labelling in cell processes located in the apical region of the OE, in some elements of the basal layer and a few cells in the nonsensory epithelium. However, regarding the OB the authors only found positive glomerular fields in five of seven trouts, and in a heterogeneous shape. Pastor et al.^101^, studied two Teleostei, *Sparus auratus* and *Dicentrachus labrax* finding negative reaction in the olfactory rosette. Our own findings highlight the interspecific diversity in UEA marking, as we have found a positive reaction on the luminal surface of the neuroepithelium and a high concentration of l-fucose in the crypts of nonsensory epithelium, probably due to the mucosal secretion of these cells. Regarding the OB we did not find labelling in our specimens.

LEA has been more widely used to characterize the olfactory system of fish than UEA, but surprisingly there are no specific studies in zebrafish, where we have seen that it is an excellent marker of the sensory epithelium. LEA labels all its cellular elements, establishing a clear border with the unlabelled nonsensory epithelium. The striking presence in the crypts of individualized cells with a clear neuronal morphology, oval shape and fine dendritic processes, has not previously described and should be object of future studies. Interestingly, the neuronal features of these cells, would coincide with the observations by Amato et al.^102^ who found TRPV4 immunoreactive “unknown cells” in the nonsensory epithelium of the zebrafish olfactory rosette. TRPV4 is a nonselective cation channel that belongs to the vanilloid subfamily of transient receptor potential ion channels. These authors suggest that these TRPV4 cells might be involved in olfactory sensation. Moreover, Parisi et al.^64^ verified that TRPV4 cells did not colocalized with calretinin, which is consistent with the lack of calretinin immunopositive cells in the crypts, reported by us. Moreover, our immunolabelling with anti-GAP-43 produced a similar pattern in the crypt epithelium when compared to LEA. All these results together point for the first time to a chemosensory nature of the crypts LEA positive cells.

All mammal and fish species studied till date have shown positivity to LEA in their olfactory sensory epithelia, apart from the case of Pleuronectiformes^103^, in which surprisingly and as an exception, LEA negative staining in the OE was reported. Regarding the OB, our results are consistent with those observed in the lungfish *Protopterus annectens*^104^ where LEA positivity was found in the ventral part of the OB, a region associated with reproductive behaviour^105^.

Our marking with BSI-B_4_ stained both the olfactory and the nonsensory epithelium, respectively producing a striking pattern containing scattered cells in the olfactory sensory epithelium, and a widespread labelling of neuronal-like cells similar to those identified with LEA in the nonsensory epithelium. There are no references to compare this striking result in zebrafish, since studies with this lectin existing in other fish species, such as eels and sharks^106,107^ have been restricted to the sensory epithelium, where these authors described a diffuse labelling.

Overall, we have performed the comprehensive characterization of the zebrafish olfactory system at the morphological, lectin, and immunohistochemical levels, providing new information for a wide range of previously unexplored markers and further confirming the characterization of previously examined markers using independent methodological approaches. Moreover, our results showed that specific markers of the transduction chain previously associated with mammalian vomeronasal receptors are also effectively expressed in the rosette and olfactory bulb of zebrafish and, additionally, suggest the possible involvement of the nonsensory zone of the olfactory rosette epithelium in chemoreception. These results significantly add to the currently available information regarding the neurochemical profile of the zebrafish olfactory system, indicating that the zebrafish system displays greater complexity that is currently acknowledged.

## Supplementary Information

Supplementary Information.
