# Supplementary material for: A comprehensive structural, lectin and immunohistochemical characterization of the zebrafish olfactory system
Source: Sci Rep. 2021 Apr 23;11:8865. doi: 10.1038/s41598-021-88317-1 (PMC8065131; doi:10.1038/s41598-021-88317-1)
Supplement: Supplementary file 1 — Supplementary Information. [file 41598_2021_88317_MOESM1_ESM.pdf]

A comprehensive Structural, Lectin, and Immunohistochemical Characterization of the Zebrafish Olfactory System

Paula R. VILLAMAYOR [a,b]; Álvaro. J. ARANA [b]; Carlos COPPEL [b]; Irene ORTIZ-LEAL [a]; Mateo V. TORRES [a]; Pablo SANCHEZ-QUINTEIRO [a*]; Laura SÁNCHEZ [b,c]

[a] Department of Anatomy, Animal Production and Clinical Veterinary Sciences. Faculty of Veterinary. University of Santiago de Compostela. Lugo, Spain.

[b] Department of Zoology, Genetics and Physical Antropology. Faculty of Veterinary. University of Santiago de Compostela. Lugo, Spain.

[c] Preclinical Animal Models Group. Health Research Institute of Santiago de Compostela (IDIS).

**Correspondence:*

Dr. Pablo Sanchez-Quinteiro. Department of Anatomy, Animal Production, and Clinical Veterinary Sciences.

Faculty of Veterinary, University of Santiago de Compostela, Av Carballo Calero s/n, 27002 Lugo, Spain.

e-mail: pablo.sanchez@usc.es

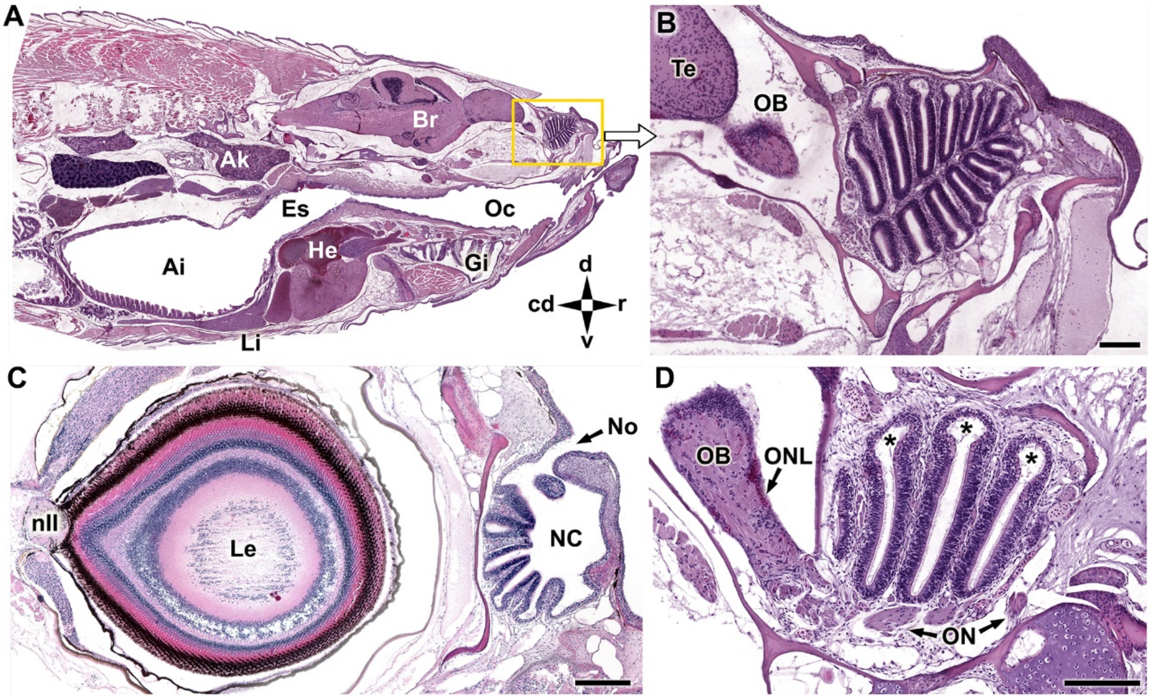

Supplementary Fig. S1 Microscopic anatomy of the zebrafish olfactory system. (A) Low power sagittal section of the anterior zebrafish body showing its main morphological features. (B) Higher magnification of the inset in A showing a general view of the olfactory rosette and the olfactory bulb (OB). (C) Lateral sagittal section of the head showing the topographical relationships of the nasal cavity with the eyeball. The dorsal nostril (No) is also shown. (D) Medial sagittal section of the olfactory rosette showing its connection with the OB through the branches of the olfactory nerve (ON). The upper rims of the lamellae form a channel-like system (asterisks). Ai, anterior intestine; Ak, anterior kidney; Br, brain; Es, esophagus; Gi, gilts; He, heart; Le, lens; Li, liver; nII, optical nerve; NC, nasal cavity; Oc, oral cavity; ONL, olfactory nerve layer; Te, telencephalon; cd, caudal; d, dorsal; r, rostral; v, ventral. Staining: Hematoxylin-eosin. Scale bars: 100 μm.
